# Suppression of *OsMADS7* in rice endosperm stabilizes amylose content under high temperature stress

**DOI:** 10.1111/pbi.12745

**Published:** 2017-05-24

**Authors:** Hua Zhang, Heng Xu, Mengjie Feng, Ying Zhu

**Affiliations:** ^1^ State Key Laboratory Breeding Base for Zhejiang Sustainable Pest and Disease Control Key Laboratory of Creative Agriculture Ministry of Agriculture Institute of Virology and Biotechnology Zhejiang Academy of Agricultural Sciences Hangzhou Zhejiang China

**Keywords:** rice, grain quality, amylose content, high temperature, seed development, MADS‐box gene

## Abstract

High temperature significantly alters the amylose content of rice, resulting in mature grains with poor eating quality. However, only few genes and/or quantitative trait loci involved in this process have been isolated and the molecular mechanisms of this effect remain unclear. Here, we describe a floral organ identity gene, *OsMADS7*, involved in stabilizing rice amylose content at high temperature. *OsMADS7* is greatly induced by high temperature at the early filling stage. Constitutive suppression of *OsMADS7* stabilizes amylose content under high temperature stress but results in low spikelet fertility. However, rice plants with both stable amylose content at high temperature and normal spikelet fertility can be obtained by specifically suppressing *OsMADS7* in endosperm. GBSSI is the major enzyme responsible for amylose biosynthesis. A low filling rate and high expression of *GBSSI* were detected in *OsMADS7* RNAi plants at high temperature, which may be correlated with stabilized amylose content in these transgenic seeds under high temperature. Thus, specific suppression of *OsMADS7* in endosperm could improve the stability of rice amylose content at high temperature, and such transgenic materials may be a valuable genetic resource for breeding rice with elite thermal resilience.

## Introduction

Global mean surface temperatures continue to increase in this century (IPCC, 2007), resulting in many negative impacts on crop production (Lesk *et al*., 2016). Rice, one of the most important cereal crops feeding more than half of the global population, is relatively sensitive to HT (high temperature). HT stress during the grain filling stage causes not only low yield but also poor quality in rice (Peng *et al*., 2004; Yamakawa *et al*., 2007). When the filling temperature is higher than 28 °C, a dramatic decrease in AC (Amylose Content) and increase in chalky appearance occur in some *Japonica* cultivars (Lyman *et al*., 2013; Sreenivasulu *et al*., 2015; Yamakawa *et al*., 2007). Abnormal starch granules were observed in the endosperm, suggesting that starch metabolism is greatly impaired under HT (Yamakawa *et al*., 2007; Zhang *et al*., 2016).

Accordingly, gene expression profiles reveal significant changes in starch metabolism pathways at HT. Most of the starch biosynthesis genes, such as *GBSSI* (*granule‐bound starch synthase I*), *SBE* (*starch branching enzyme*) and *cyPPDK* (*cytosolic pyruvate orthophosphate dikinase*), are down‐regulated, while some starch hydrolysing genes, such as α‐amylases (*Amy1A*,* Amy1C*,* Amy3A*,* Amy3D* and *Amy3E*), are induced under HT in developing seeds (Hakata *et al*., 2012; Liao *et al*., 2015; Wang *et al*., 2015; Yamakawa *et al*., 2007). It has been suggested that the alteration of expression in these starch metabolism‐related genes in rice might be the cause of poor grain quality under high filling temperatures (Cheng *et al*., 2005; Yamakawa *et al*., 2007). Overexpression of *Amy1A* or *Amy3D* results in more chalky grains even under normal growth conditions, while suppression of some α‐amylase genes in the endosperm could improve rice quality by reducing grain chalkiness at HT (Hakata *et al*., 2012). Thus, down‐regulation of α‐amylase expression could be a potential strategy for ameliorating grain chalkiness caused by HT at the grain filling stage. However, suppressed expression of α‐amylase genes had no effect on AC in rice seeds (Hakata *et al*., 2012).

AC, as an important physicochemical property for rice eating and cooking quality, is mainly determined by GBSSI (Liu *et al*., 2014; Sano, 1984; Wang *et al*., 1995). Previous data showed that the *Wx* gene encoding *GBSSI* was down‐regulated at HT, which led to a considerable reduction in AC (Lin *et al*., 2005; Yamakawa *et al*., 2007; Zhang *et al*., 2014). In addition, *SBE* also plays important roles in amylose biosynthesis (Zhu *et al*., 2012). Overexpression of *Wx* and down‐regulation of *SBE* both result in very high AC in rice seeds, while suppression of *GBSSI* expression leads to extremely low AC (Liu *et al*., 2005; Tian *et al*., 2009; Zhu *et al*., 2012). As modest AC (15%~25%) is a significant index for high‐quality rice, simply overexpressing *GBSSI* or suppressing *SBE* may not be suitable for rice breeding. Therefore, fine‐tuning of starch metabolic genes might be an alternative strategy for high‐quality rice breeding in a globally warming climate.

Expression of the *Wx* gene can be regulated at multiple levels. Several transcription factors involved in *Wx* expression have been identified. OsBP‐5 and OsEBP‐89 were found to synergistically regulate the transcription of the *Wx* gene. Disturbing OsBP‐5 expression by RNAi resulted in a mild reduction in AC in transgenic seeds (Zhu *et al*., 2003). *OsbZIP58* is another endosperm‐specific transcription factor involved in the regulation of both the *Wx* and *SBEI* genes. A null mutant *osbzip58* showed decreased AC and altered seed morphology (Wang *et al*., 2013). Furthermore, expression of the *Wx* gene is finely controlled at the post‐transcriptional level. Alteration of *Wx* splicing efficiency by a SNP at the splicing site (Cai *et al*., 1998) or mutations in the spliceosome (Isshiki *et al*., 2006; Zeng *et al*., 2007) could affect its mRNA abundance, which in turn results in reduced AC in their seeds. Our recent data showed that four major quantitative trait loci (QTLs) from *Indica* cultivar 9311 could reduce the deleterious effects of high temperature on the AC of the *Japonica* cultivar Nipponbare by increasing the splicing efficiency of *Wx* pre‐mRNA (Zhang *et al*., 2014). However, no genes have yet been reported to maintain a stable AC at HT during the ripening stage.

The MADS‐box genes belong to a large transcription factor family including 75 members in the rice genome (Arora *et al*., 2007). Most of them were found to be essential for floral organ identity (Li *et al*., 2011; Liu *et al*., 2013). Some play critical roles in rice endosperm development. For instance, *OsMADS29* was presumed to be a key regulator of rice seed development (Nayar *et al*., 2014; Yang *et al*., 2012; Yin and Xue, 2012). *OsMADS87* was found to be involved in endosperm cellularization and the regulation of seed size (Chen *et al*., 2016). Moreover, some MADS‐box members also play important roles in stress responses. *OsMADS26* negatively controls plant resistance to pathogens and drought stress (Khong *et al*., 2015). A set of type I MADS‐box genes including Os*MADS87* show responses to heat stress during the early stage of endosperm development (Chen *et al*., 2016).

*OsMADS7* (LOC_Os08 g41950), an important transcription factor regulating floral identity, is highly expressed in rice reproductive organs, such as spikelet meristems, developing lodicules, stamens and pistils (Cui *et al*., 2010). Based on gene expression data (http://rice.plantbiology.msu.edu), *OsMADS7* is also expressed abundantly at the early and middle milk stages (Kawahara *et al*., 2013). However, the biological function of *OsMADS7* in seed development remains unknown. Here, we found that *OsMADS7* can be highly induced by HT in rice endosperm. Plants with suppression of *OsMADS7* exhibited less reduction in AC than WT under HT, suggesting a potential role of *OsMADS7* in heat stress response in rice seeds. Specific down‐regulation of *OsMADS7* in endosperm which avoiding the reduction in fertility could be a potential strategy for improving the thermal resilience of rice for better quality and stable yield.

## Results

### Expression of *OsMADS7* in rice endosperm is induced by HT

**Figure 1:**
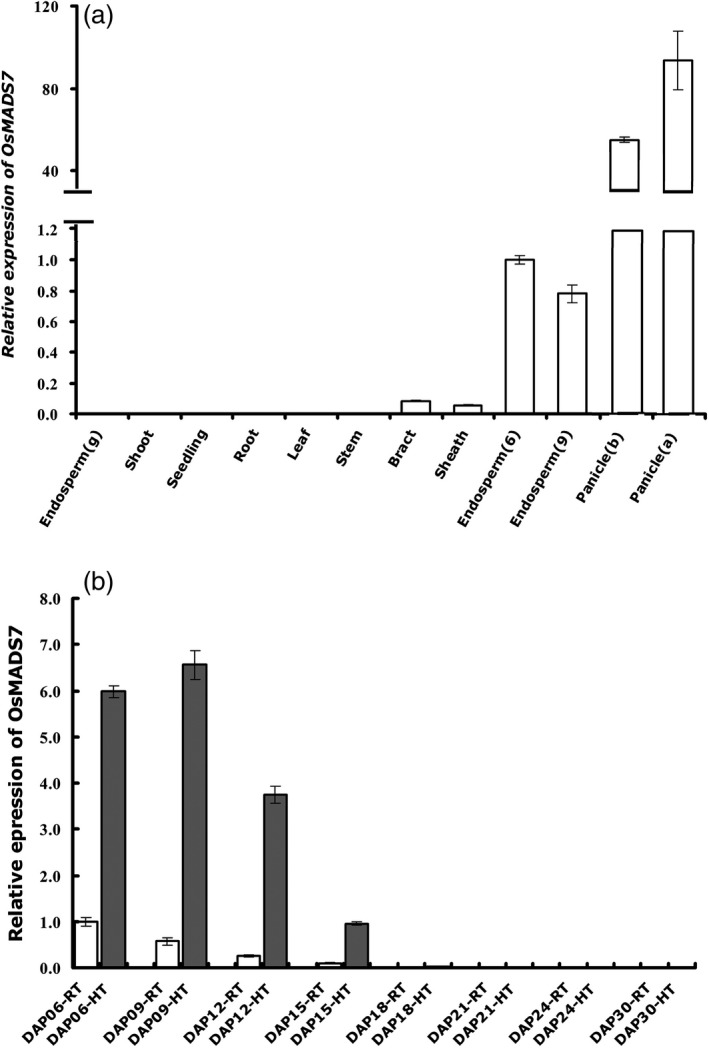
*OsMADS7* is expressed intermediately in rice endosperm and induced by HT at the milk stage. Transcripts were measured using qRT‐PCR and normalized to the level of the control sample (6 DAP endosperm) using *UBQ10* as internal control. (a) Expression pattern of *OsMADS7* in different tissues. Endosperm (g) indicates endosperm at the seed germination stage; endosperms (6 and 9) indicate developing endosperm at 6 and 9 DAP at the filling stage; panicle (b and a) indicate panicles before and after flowering, respectively. (b) Expression of *OsMADS7* in developing endosperm is induced by HT. Data represent means ± SE, *n* = 3 biological replicates, 6–10 developing seeds in each replicate. DAP: day after pollination. H and R indicate seeds harvested from HT and RT growth conditions, respectively.

To isolate more regulators involved in seed quality control at HT, the transcription factor highly expressed in rice endosperm (Fu and Xue, 2010) was selected and subjected to expression analysis under long‐term HT treatment at milk stage as described before (Xu *et al*., 2015). *OsMADS7* is one of the HT responsive transcription factors screened out from above assay. According to a public database (RGAP, www.rice.plantbiology.msu.edu), published data (Kawahara *et al*., 2013) and our RNA‐seq data (Xu *et al*., 2015), *OsMADS7* is abundantly expressed in floral organs (inflorescence, anther and pistil), seed and endosperm. To further verify the expression pattern of *OsMADS7*, different vegetative tissues and reproductive tissues were analyzed by quantitative RT‐PCR assay. As expected, *OsMADS7* showed the highest expression in panicles and intermediate expression in endosperm (Figure 1a). To further examine whether *OsMADS7* expression could be altered by HT stress at the milk stage, Nipponbare rice plants (*Oryza sativa* L. *Japonica*) were either subjected to HT treatment (day/night temperature of 35 °C/28 °C) or were maintained at RT (room temperature, day/night temperature of 28 °C/22 °C) at three DAP (day after pollination). Developing endosperm from different growth temperatures were collected at 6, 9, 12, 15, 18, 21, 24 and 30 DAP. As shown in Figure 1b, *OsMADS7* was expressed mainly in early development stages in rice endosperm (6, 9, 12 and 15 DAP), and its expression level increased more than sixfold at HT.

### Seed quality assay

**Figure 2:**
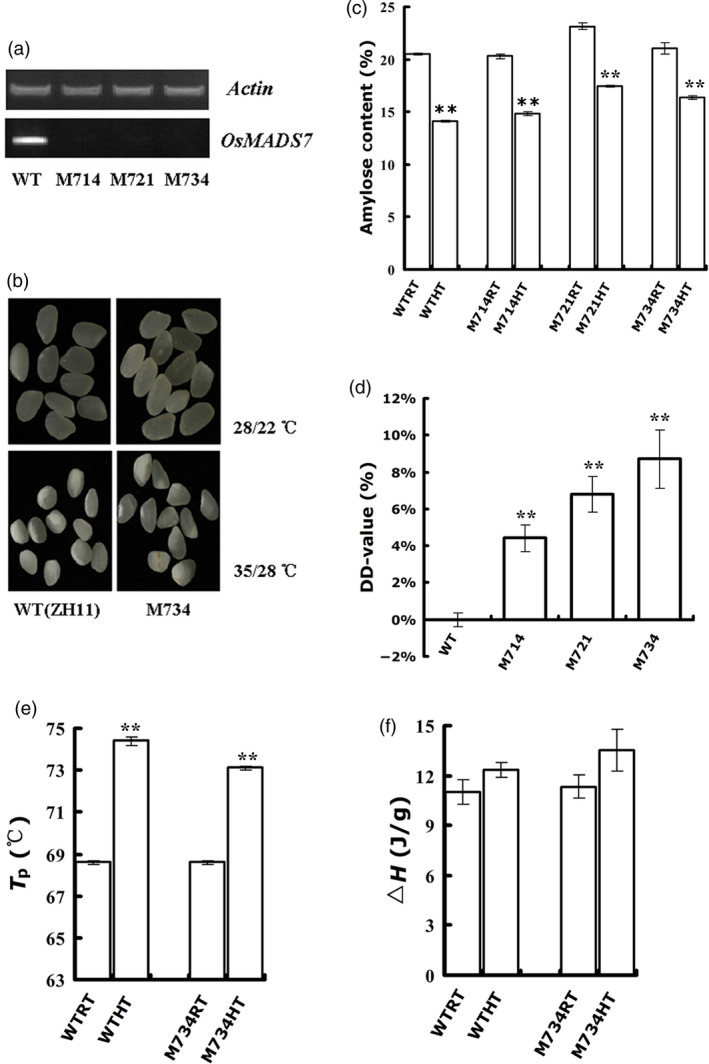
Quality analysis of *OsMADS7* RNAi rice lines with constitutive suppression. (a) Expression of *OsMADS7* in the endosperm (8 DAP) of RNAi lines and WT plants. (b) Appearance of polished rice of RNAi line M734 and WT. (c) The AC of WT (ZH11) and RNAi line seeds grown at different temperatures. (d) The DD‐value of RNAi lines. The D‐value of WT was set to zero and used as a control. (e, f) Rice Tp and Δ*H* from WT and M734. Tp: peak transition temperature; Δ*H*: enthalpies of gelatinization. In (c‐f), data represent means ± SE, *n* = 3 biological replicates. A significant difference was determined by Student's *t*‐test, not significant (N.S.), *P*‐value <0.01(**).

To test the biological function of *OsMADS7* in endosperm development, especially under the HT condition, independent constitutive RNAi lines of *OsMADS7* were obtained from the Chinese Academy of Sciences, Beijing (Cui *et al*., 2010). Three lines with constitutive suppression of *OsMADS7* were selected in this study (Figure 2a) and named M714, M721 and M734. After HT treatment, mature seeds from these lines and their WT counterpart (ZH11, *O. sativa* L. *Japonica*) were harvested for quality measurements (Figure 2b).

The AC from WT and *OsMADS7* RNAi lines decreased significantly at HT (Figure 2c). However, the AC in RNAi seeds was slightly higher than in the WT seeds at HT, especially in M721 and M734 seeds (Figure 2c). As we mentioned in Zhang *et al*. (2014) and in the Materials and methods, the DD‐value was used to measure AC stability at HT. A greater DD‐value indicates higher stability. As shown in Figure 2d, DD‐values of all three RNAi transgenic seeds were positive, suggesting that the AC of transgenic seeds is more stable than that of WT under HT. Thus, suppression of *OsMADS7* expression could ameliorate the reduction in AC caused by HT.

Starch GT (gelatinization temperature) is another important physicochemical index of rice quality. To determine whether *OsMADS7* has any effect on GT under HT, we conducted a DSC (differential scanning calorimetry) analysis between the M734 line (the line with highest DD‐value) and WT (Figure 2d). Consistent with a previous report (Zhang *et al*., 2016), the Tp (peak transition temperature, Figure 2e) and Δ*H* (gelatinization enthalpies, Figure 2f) of rice starch were increased under HT, which suggests that the GT of rice seeds grown at HT is elevated. Δ*H* primarily reflects the loss of double helical order and its value is mainly determined by proportion of amylose double helices and AC value (Cooke and Gidley, 1992; Matveev *et al*., 2001). Increase in GT and Δ*H*, together with the reduction in AC, implies that the structure of amylopectin might be changed and the proportion of amylose double helices should increase at HT. The significantly higher AC (*P* < 0.001, Figure 2c) and similar or slightly higher Δ*H* value (*P* = 0.26, not significant, Figure 2f) of M734 than that of WT suggested amylose double helices in M734 might be higher than that of WT.

Moreover, GT is mostly affected by amylose starch, amylose double helices and the B‐type crystalline form of amylopectin (Richardson *et al*., 2000; Shi *et al*., 1998). M734 got significantly lower Tp (*P* < 0.01, Figure 2e) but higher AC than WT at HT suggesting M734 is different from WT in terms of amylopectin structure and the proportion of B‐type crystalline form of amylopectin in M734 might be lower than that of WT.

### Grain filling dynamics

**Figure 3:**
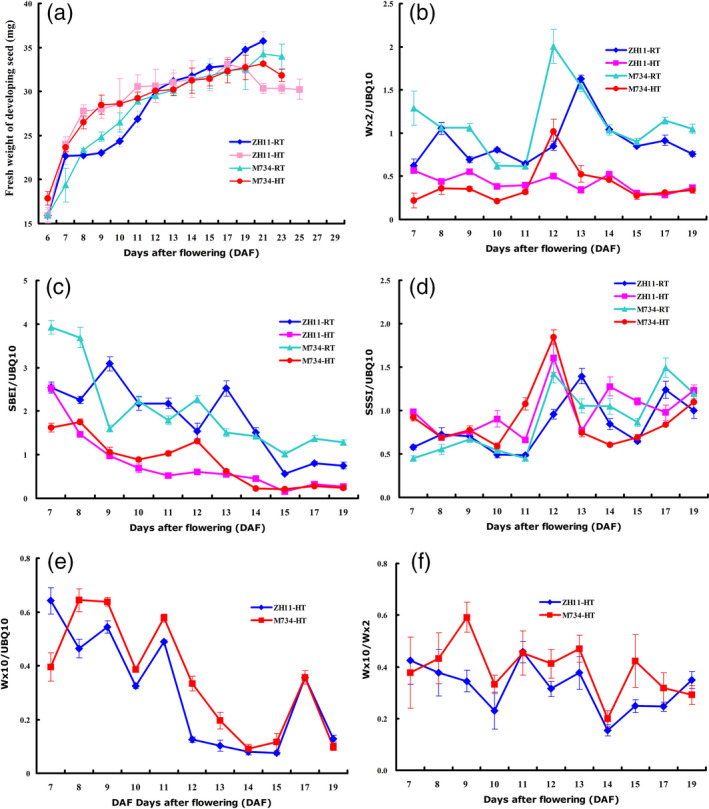
Grain filling rate and gene expression profiles from developing seeds at different temperatures. (a) Grain filling rate of RNAi line M734 and WT (ZH11) under HT and control (RT) treatment conditions. (b–d) Related expression levels of *GBSSI* (Wx2), *SBEI* and *SSSI* to *UBQ10* in developing seeds under HT and RT conditions. (e, f) Intensity of mature *Wx* transcript (Wx10) and splicing efficiency (Wx10/Wx2) of *GBSSI* in developing seeds from WT (ZH11) and M734 at HT.

To better understand the change in rice quality caused by the alteration of *OsMADS7* expression, a dynamic study of grain filling rate was carried out in the M734 and WT lines at different temperatures. As shown in Figure 3a, both genotypes had a higher grain filling rate at HT than at RT during the early filling stage (7–12 DAP), which might be the cause of impaired rice quality under HT. However, the difference in grain filling rate between HT and RT is smaller in M734 than that in WT, especially during the early filling stage (6–10 DAP) (Figure 3a); this might be the reason for relatively stable AC in M734 under HT.

As many starch synthase genes may be modulated by HT (Yamakawa *et al*., 2007), a dynamic expression analysis of starch biosynthesis genes was carried out during the filling stage. Most of the genes tested, such as *GBSSI* (*Wx*), *SBEI* (Figure 3b, c), *SBEIIb*,* AGP2b*,* AGP2L* and *AGP3L* (Figure S1), were suppressed by HT during the entire filling process. *SSSI* (Figure 3d) is the only gene whose expression was slightly induced by HT. When considering genetic background, *GBSSI* (*Wx*), *AGP2L* and *AGP3L* showed different expression patterns between the M734 RNAi line and WT, while *SBEI*,* SBEIIb*,* SSSI* and *AGP2b* showed similar expression patterns between them. Although both *AGP2L* and *AGP3L* showed lower expression levels in M734 at RT than those of the WT, *AGP3L* showed higher level at HT than those of the WT, while *AGP2L* showed no big difference with WT (Figure S1). Moreover, the expression intensity of *GBSSI (Wx)* was higher in M734 than in WT at peak point (12 or 13 DAP) under both temperature conditions (Figure 3b).

Post‐transcriptional regulation is one of the most important mechanisms for modulation of *Wx* expression (Cai *et al*., 1998). As we mentioned in Zhang *et al*. (2014), the splicing efficiency of the first intron of *Wx* is represented by the ratio of Wx10 (mature *Wx* transcript) to Wx2 (total *Wx* transcript). qRT‐PCR results revealed that both the splicing efficiency of the first intron and the mature transcript products of *Wx* gene were higher in M734 than in WT under HT during almost the entire filling stage (Figure 3e, f), which might result in high AC and partially explain the stability of AC in *OsMADS7* RNAi lines under HT. Therefore, our results suggest that suppression of *OsMADS7* causing improved AC stability under HT might be mediated through regulation of *Wx* expression.

### Suppression of *OsMADS7* expression affects rice spikelet fertility

**Figure 4:**
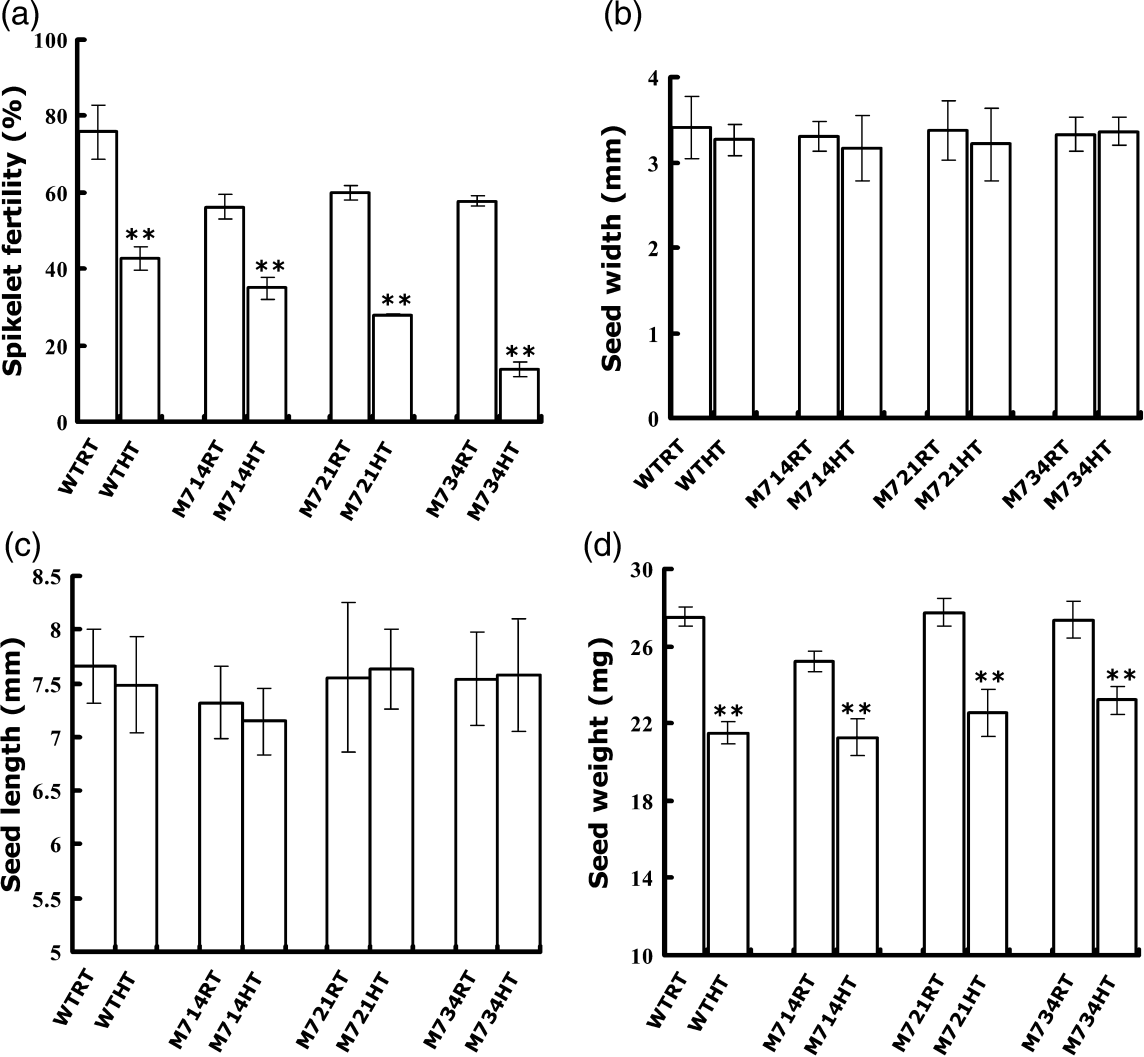
Morphologic analysis of rice seeds from constitutively suppressed *OsMADS7* lines. (a) spikelet fertility (*n* = 5), (b) seed width, (c) seed length and (d) seed weight (*n* ≥ 300) of suppression lines of *OsMADS7* and WT (ZH11) under high temperature (HT) and control (RT) treatments. Data represent means ± SE. A significant difference was determined by Student's *t*‐test, *P*‐value <0.01(**).

*OsMADS7* is an important transcription factor involved in flower development. Constitutively knocking down of this gene resulted in low spikelet fertility in rice (Cui *et al*., 2010). To test the effect of high temperature on this process, plants were moved into chambers at the booting stage. Consistent with a previous report (Cui *et al*., 2010), all three *OsMADS7* RNAi lines (M714, M721 and M734) displayed lower spikelet fertility (56%~60%) than that of WT (76%) at the control temperature. High temperature showed an additive effect. Under HT, the fertility of the *OsMADS7* RNAi lines was further decreased to 13.8%~34.9%, while it was decreased only 42.7% in WT (Figure 4a). These data indicate that constitutive down‐regulation of *OsMADS7* expression also results in low fertility. Additionally, we found that HT affected seed width (Figure 4b) and length (Figure 4c) slightly and reduced the seed weight significantly (*P* < 0.001, Figure 4d). However, no significant difference in seed size was found between RNAi lines and WT (Figure 4b–d). All results indicated that constitutive suppression of *OsMADS7* led to reduced spikelet fertility under HT, which is not suitable for breeding plants with better quality.

### Endosperm‐specific down‐regulation of *OsMADS7* increases the stability of rice AC at HT

Low spikelet fertility in *OsMADS7* RNAi lines may be due to the down‐regulation of *OsMADS7* in floral organs. To avoid this effect, we generated a set of endosperm‐specific RNAi lines by using a rice endosperm‐specific promoter from *GluC* (LOC_Os02g25860) (Qu *et al*., 2008).

**Figure 5:**
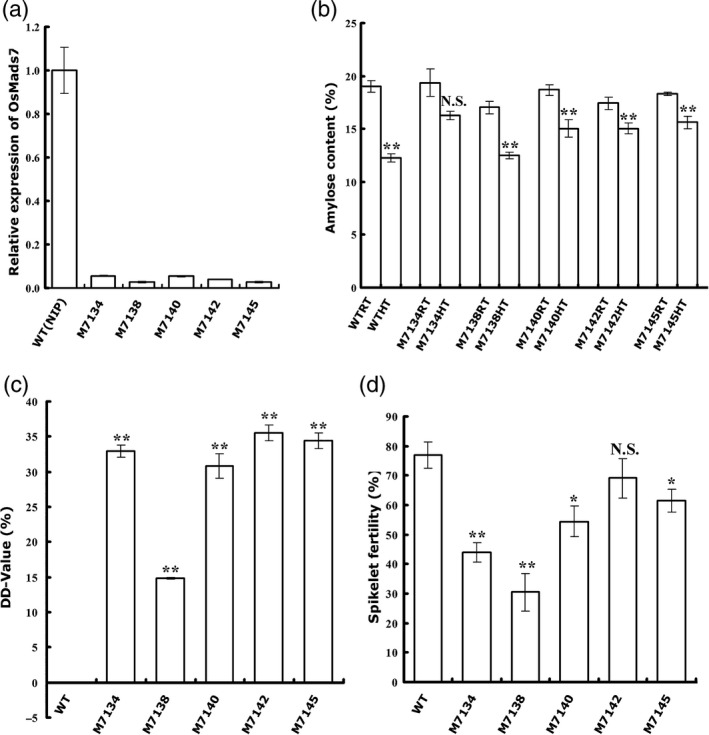
Characterization of endosperm‐specific suppression lines of *OsMADS7*. (a) Relative expression of *OsMADS7* in rice endosperm (9 DAP) in the suppression lines and WT (NIP). Transcripts were measured using qRT‐PCR and normalized to the level of WT control using *UBQ10* as internal control. Data represent means ± SE, *n* = 3 biological replicates. (b) Rice AC of the suppression lines and WT at high temperature (HT) and room temperature (RT). (c) DD‐values of the suppression lines between the HT and RT conditions. (d) Spikelet fertility of suppression lines of *OsMADS7* and WT under HT conditions. Data represent means ± SE, *n* = 5 biological replicates. A significant difference was determined by Student's *t*‐test, not significant (N.S.), *P*‐value <0.05(*), *P*‐value <0.01(**).

Nineteen independent T_0_ RNAi lines (M7130–7148) were generated and the expression levels of *OsMADS7* in the developing seeds at 9 DAP were examined. No obvious developmental deficiency was observed in these lines. Five transgenic lines (M7134, 7138, 7140, 7142 and 7145) with more than 17‐fold down‐regulation of *OsMADS7* (less than 6% of WT, Figure 5a) in rice endosperm were selected, and their T_2_ plants were used for HT treatment. Mature seeds from these RNAi lines and a WT control (Nipponbare, *O. sativa* L. *Japonica*) were collected for AC measurement. The results showed that all the transgenic seeds had a similar AC to WT at RT. However, the AC from WT seeds was decreased approximately 35.6% at HT, while the reduction in AC in the RNAi seeds was much smaller (16.2%, 26.6%, 19.5%, 13.7% and 14.6%, Figure 5b). Additionally, one line (M7134) showed no significant difference in AC between HT and RT (*P* = 0.06, Figure 5b). The DD‐values of these RNAi lines ranged from 14% to 36% (Figure 5c). Four lines (M7134, 7140, 7142 and 7145) had DD‐values above 30%, higher than any line with a constitutive RNAi construct (Figure 2b). These data suggested that endosperm‐specific down‐regulation of *OsMADS7* was more tolerant to HT in terms of AC stability.

Spikelet fertility of endosperm‐specific RNAi lines was further examined at HT. Compared to WT (76.9 ± 4.4%), lines M7134, 7138, 7140 and 7145 showed low fertility (from 30% to 60%), while the M7142 line (69.1 ± 6.7%) displayed only a slight decrease (*P* = 0.31, Figure 5d). Interestingly, the M7142 line also had the highest DD‐value (35.5 ± 1.1%) among the five transgenic lines (Figure 5c). Similar results were obtained from the T_3_ generation (data not shown). Therefore, genetic materials that produce better rice quality at HT without significant fertility problems can be obtained by knocking down *OsMADS7* in an endosperm‐specific manner.

Thus, specific suppression of *OsMADS7* in rice endosperm is a potential strategy to increase the stability of rice AC at HT. The transgenic line M7142 might be a good genetic resource for thermal resilience rice breeding.

## Discussion

HT during the filling stage may greatly impair rice seed quality, especially in *Japonica* varieties that are more sensitive to HT (Yamakawa *et al*., 2007; Zhang *et al*., 2016). The deleterious effects manifest mainly in two aspects: increase in chalky appearance and decrease in AC (Hakata *et al*., 2012; Larkin and Park, 1999). Previous studies showed that suppression of α‐amylose genes reduces the amount of chalky grains under HT stress (Hakata *et al*., 2012). Thus, it might be a potential strategy to improve rice quality under global warming conditions. However, no genes or strategies have been reported to stabilize AC at HT without deleterious side effects. This study identified a transcription factor, *OsMADS*7 involving in AC regulation at HT. *OsMADS*7 belongs to the class E of floral organ identity genes and is highly expressed in floral organs (Cui *et al*., 2010). Constitutive silencing of *OsMADS*7 resulted in weak developmental deficiency including one carpel with three stigmas and low spikelet fertility. In this study, we confirmed that *OsMADS*7 is moderately expressed in rice endosperm and found that it can be highly induced by HT stress at the early grain filling stage. Furthermore, RNAi‐mediated suppression of *OsMADS*7 produced a relatively stabilized rice AC under HT stress, suggesting that *OsMADS*7 may also play an important role in grain filling. In addition to impaired rice quality, HT during the reproductive stage can particularly affect rice yield. In this study, we found that the spikelet fertility of *Japonica* cultivars ZH11 and Nipponbare was significantly decreased under HT stress. ZH11 (42.7%, Figure 4a) seems to be more sensitive to HT than Nipponbare (76.9%, Figure 5d) in terms of spikelet fertility. Moreover, we also evaluated the effect of HT on rice seed size and weight. Seed weight was significantly reduced under HT, while the seed length and width did not change significantly (Figure 4b–d). The results further suggest that HT produces a negative effect on storage material accumulation during seed filling. Consistent with previous reports (Yamakawa *et al*., 2007; Zhang *et al*., 2016), lower AC, higher GT and more chalky grains were found in rice seeds grown at HT than at RT conditions in this study (Figure 2b–f). Dynamic analysis (Figure 3a) further revealed that the grain filling rate of rice seed was higher under HT than that under RT at early stages (6–12 DAP). The fresh weight of WT seeds peaked much earlier at HT (17 DAP) than at RT (≥21 DAP), while that of the RNAi seeds (M734) peaked at 21 DAP under both temperature conditions (Figure 3a). These results indicated that RNAi seeds had higher grain filling rate than WT at late milky stage under HT, which may also explain the stabilized AC in RNAi lines at HT.

As starch is the main content in rice seeds, expression patterns of many starch biosynthesis genes under different temperatures were examined in a dynamic manner. Except for *SSSI*, most of the starch biosynthesis genes were down‐regulated under HT. As in Hakata's reports (Hakata *et al*., 2012), starch hydrolysing enzyme was greatly up‐regulated at HT. We deduced that the high filling rate and the alteration of gene profiles involved in starch metabolism might be the major causes of poor rice quality under HT.

It is well documented that the reduction in rice AC might be attributed to the down‐regulation of *GBSSI* (*Wx*) in developing rice seeds. Previous studies also showed that HT can modulate *Wx* expression at both transcriptional and post‐transcriptional levels (Larkin and Park, 1999; Zhang *et al*., 2014). To determine the possible mechanism of *OsMADS*7 in AC regulation at HT, dynamic analyses of starch biosynthesis gene expression were performed in the *OsMADS*7 RNAi line M734 and its WT counterpart ZH11. Both the total *Wx* transcript and the mature *Wx* transcript were higher in M734 than in ZH11 at HT (Figure 3b, e, f), suggesting that *OsMADS*7 may negatively regulate *Wx* expression at both transcriptional and post‐transcriptional levels. The stable AC in *OsMADS*7 RNAi seeds at HT might be due to the high expression of the *Wx* gene. How *OsMADS*7 participates in *Wx* gene regulation needs to be clarified in the future.

Consistent with Cui's observation (Cui *et al*., 2010), constitutive suppression of *OsMADS*7 resulted in weak phenotypes including low fertility (~60%, Figure 4a). When plants are grown at HT, the spikelet fertility of the *OsMADS7* RNAi line is decreased (<35%, Figure 4a). Thus, this kind of transgenic material may not be suitable for rice breeding, although it may improve rice quality at HT. As *OsMADS7* plays an important role in floral organ development, we speculated that the low spikelet fertility might be due to down‐regulation of *OsMADS7* in floral organs. Therefore, using an endosperm‐specific promoter to drive the suppression might be the best alternative. Indeed, *OsMADS7* was down‐regulated mainly in rice endosperm and very slightly in floral organs (Figure S2), using the new strategy. Although some transgenic lines also exhibited low spikelet fertility, others, such as M7142, were found to have the same spikelet fertility as the WT (Nipponbare) at HT (Figure 5d). *OsMADS8*, showing functional redundancy with *OsMADS7* (Cui *et al*., 2010), was up‐regulated in these transgenic lines (Figure S3), which may partially compensate the deficiency of spikelet fertility. It will be interesting to further check the insertion site of the RNAi construct or the possibility of the expression of other *OsMADS7* homologs altered in these RNAi lines to elucidate the different fertility levels between M7142 and other RNAi lines. Moreover, the DD‐values from these endosperm‐specific RNAi lines (14%–36%, Figure 5) were higher than that of constitutive RNAi lines (4.4%–8.7%, Figure 2), suggesting that endosperm‐specific suppression of *OsMADS7* might be more tolerant of HT in terms of AC stability. Thus, *OsMADS7* is a valuable candidate gene for maintaining a stable AC in rice at HT. Specific down‐regulation of *OsMADS7* in rice endosperm should be an effective strategy to breed HT‐tolerant rice with high eating quality in the future.

## Experimental procedures

### Plant materials and growth conditions

Rice varieties Nipponbare and ZH11 (*O. sativa* L. *Japonica*) were used in this study. Rice plants were grown in the experimental fields of Zhejiang Academy of Agricultural Sciences, Hangzhou, Zhejiang Province. At the initial heading stage, rice plants were transplanted into pots and then transferred to growth chambers for HT or control treatment. The HT condition in this study was 35 °C, 12‐h light/28 °C 12‐h dark; the control condition was set as 28 °C, 12‐h light/22 °C 12‐h dark.

### Phenotyping of rice seed

Spikelet fertility, the ratio of filled grains versus total spikelets, was calculated from five panicles. Morphology of the rice seed (*n* ≥ 300), such as length and width, was measured using the SC‐I test system (Wseen, China).

### Expression analysis of *OsMADS7*

More than 100 dehulled rice seeds with no disease spots were selected and incubated in distilled water in a chamber (28 °C, 12‐h light/22 °C 12‐h dark). 5 days after soaking, endosperm (g) and shoots were dissected. Endosperm (g) from 6 to 8 germinated seeds and shoots from approximately 20 germinated seeds were selected in each replicate, respectively. Seedling, root and stem were harvested at three‐leaf stage, and three plants were used in each replicate. Panicles before/after flowering were harvested from three rice plants. More than 10 developing rice seeds in each replicate were harvested at various DAPs, as the rice caryopses were marked on the initiation of pollination. Total RNAs were extracted from above tissues using TRIzol reagent (Invitrogen, USA) and reverse transcribed into cDNA with the GoScript™ Reverse Transcription System (Promega, USA) as previously described (Zhang *et al*., 2014). For RT‐PCR analysis, the rice *actin* (She *et al*., 2010) gene was used as an internal control and gene‐specific primers MADS7C2‐F1&R1 were used to analyze the expression of *OsMADS7*. qRT‐PCR assays were performed with iQ™ SYBR^®^ Green Supermix (Bio‐Rad, USA) on a CFX96™ Real‐Time System (Bio‐Rad) with *UBQ10* (Jain *et al*., 2006) as the reference gene. Primer pairs MADS7C2‐F5&R5 and MADS8‐F3&R3 were used to specifically identify transcripts of *OsMADS7* and *OsMADS8*, respectively, in the qRT‐PCR assays (Table S1). All experiments were repeated three times. Data are means ± SE (*n* = 3).

### Analysis of rice quality

Mature seeds were dried in the 39 °C oven for at least 5 days. Rice seeds (*n* = 48) were then cut into two parts. The part with embryo was germinated in water, and the other part was used for rice quality analysis. Seven days after germination, genomic DNAs were extracted from germinating part and verified with primer pair HYG‐F&R. Positive and negative seeds were selected as transgenic and control (WT) samples, respectively.

Before rice quality analysis, the starch was purified from polished rice using the alkaline protease method (Zhu *et al*., 2010). Rice AC was measured using an iodine colorimetric method (Juliano, 1971) with slight modification (Zhang *et al*., 2014). The *D*‐value of WT seeds was set to zero and the AC of RNAi lines was represented by the difference (*D*‐value = (AC_RNAi_ − AC_WT_)/AC_WT_) between the transgenic lines and WT. For each transgenic line, two *D*‐values (*D*_RT_ and *D*_HT_) were obtained. The difference in the *D*‐values (*D*_HT_ − *D*_RT_) between *D*_HT_ and *D*_RT_, named as DD‐value, was used to display the AC stability of the transgenic lines under HT. Starch gelatinization and retrogradation temperatures were measured by a differential scanning calorimeter (DSC 200F3, Netzsch Instruments NA LLC, Burlington, MA, USA) as previously described (Zhang *et al*., 2013).

### Generation of *OsMADS7* endosperm‐specific knockdown transgenic plants

To construct an endosperm‐specific RNAi vector, the promoter of the *GluC* (2331 bp) (Qu *et al*., 2008) was amplified by PCR with primer pair GluCp‐1F&1R. The promoter was then inserted into the *HindIII* and *PstI* restriction sites of the vector pCAMBIA1301. Subsequently, the rice intron from vector PTCK303 (Wang *et al*. 2004) was fused into the above vector at restriction sites *PstI* and *SacI* to yield the endosperm‐specific RNAi vector pGluc‐RNAi. The specific fragment of *OsMADS7* used for the RNAi construct was amplified with primer pair MADS7C2‐F6&R6. Then, the fragment of *OsMADS7* was cloned into two sides of the rice intron with restriction enzymes *SalI* and *PstI*,* SacI* and *SpeI*, respectively, resulting in the final *OsMADS7* endosperm‐specific RNAi vector (GluC‐MADS7‐RNAi). The RNAi vector was introduced into Agrobacterium strain EHA105, and rice transformation was performed as previously described (Hiei *et al*., 1994).

### Dynamic analysis of developing rice seeds

At the heading stage, more than 30 rice plants from transgenic line M734 or WT were transplanted from the field into pots. Caryopses were marked on the initiation of pollination. At 3 DAP, rice plants were transferred to chambers for HT or RT treatment. Thirty developing seeds were harvested at each time point (6–15, 17, 19, 21, 23, 25 and 27 DAP). The fresh weight was measured for dynamic filling rate analysis. The seeds were then immediately frozen in liquid nitrogen and stored at −80 °C for dynamic analysis of gene expression. Specific primers were used in the dynamic expression analysis for starch biosynthesis genes (Table S1).

## Conflict of interest

The authors declare no conflict of interest.

## Supporting information


**Figure S1** Expression of starch biosynthesis genes in developing seeds grown at different temperatures. Related expression levels of *SBEIIb, AGP2L, AGP3L* and *AGP2b* to *UBQ10* in developing seeds under HT and RT conditions.
**Figure S2** Relative expression of *OsMADS7* in rice panicles of endosperm‐specific suppression lines and WT (NIP). Transcripts were measured using qRT‐PCR and normalized to the level of WT control using *UBQ10* as internal control. Data represent means ± SE, *n* = 3 biological replicates.
**Figure S3** Relative expression of *OsMADS8* in rice panicles of endosperm‐specific suppression lines of *OsMADS7* and WT (NIP). Transcripts were measured using qRT‐PCR and normalized to the level of WT control using *UBQ10* as internal control. Data represent means ± SE, *n* = 3 biological replicates.
**Table S1** Oligonucleotide primer sequences used in this study.
