# Supplementary material for: Suppression of OsMADS7 in rice endosperm stabilizes amylose content under high temperature stress
Source: Plant Biotechnol J. 2017 May 24;16(1):18–26. doi: 10.1111/pbi.12745 (PMC5785353; doi:10.1111/pbi.12745)
Supplement: Supplementary file 1 — Figure S1 Expression of starch biosynthesis genes in developing seeds grown at different temperatures. Related expression levels of SBEIIb, AGP2L, AGP3L and AGP2b to UBQ10 in developing seeds under HT and RT conditions. Figure S2 Relative expression of OsMADS7 in rice panicles of endosperm‐specific suppression lines and WT (NIP). Transcripts were measured using qRT‐PCR and normalized to the level of WT control using UBQ10 as internal control. Data represent means ± SE, n = 3 biological replicates. Figure S3 Relative expression of OsMADS8 in rice panicles of endosperm‐specific suppression lines of OsMADS7 and WT (NIP). Transcripts were measured using qRT‐PCR and normalized to the level of WT control using UBQ10 as internal control. Data represent means ± SE, n = 3 biological replicates. Table S1 Oligonucleotide primer sequences used in this study. [file PBI-16-18-s001.doc]

**Supplementary files**


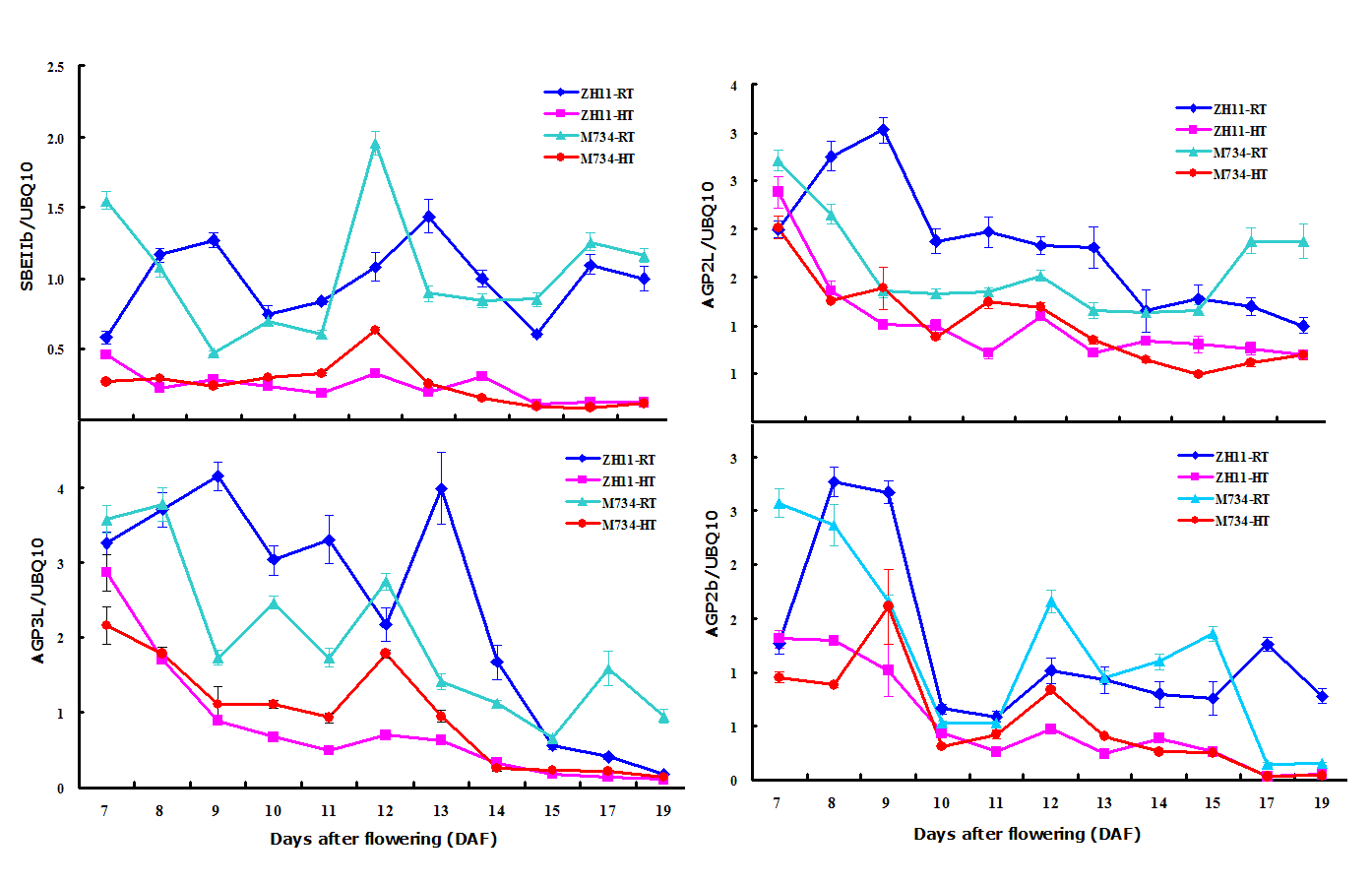


**Figure S1.** Expression of starch biosynthesis genes in developing seeds grown at different temperatures. Related expression levels of *SBEIIb，AGP2L*，*AGP3L* and *AGP2b* to *UBQ10* in developing seeds under HT and RT conditions.


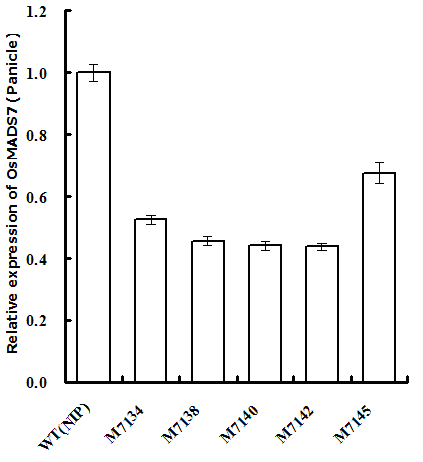


**Figure S2.** Relative expression of *OsMADS7* in rice panicles of endosperm-specific suppression lines and WT (NIP). Transcripts were measured using qRT-PCR and normalized to the level of WT control using *UBQ10* as internal control. Data represent means ± SE, n = 3 biological replicates.


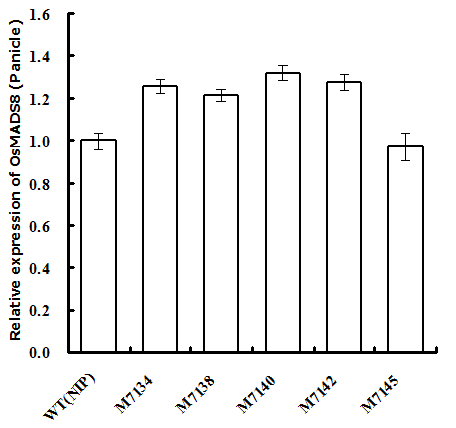


**Figure S3.** Relative expression of *OsMADS8* in rice panicles of endosperm-specific suppression lines of *OsMADS7* and WT (NIP). Transcripts were measured using qRT-PCR and normalized to the level of WT control using *UBQ10* as internal control. Data represent means ± SE, n = 3 biological replicates.

**Table S1.** Oligonucleotide primer sequences used in this study.

| Primers | DNA Sequence (5’—3’) |
| --- | --- |
| GluCp-1F | GGGAAGCTTGTTCAAGATTTATTTTTGG |
| GluCp-1R | ACGCCTGCAGAGTTATTCACTTAGTTTCCC |
| MADS7C2-F1 | CAGTTACGCAGGACCCGAA |
| MADS7C2-R1 | ACGCACTGTTCATCGCCTC |
| MADS7C2-F5 | CAGCACCCAGAGCATGACTA |
| MADS7C2-R5 | CGGCTAGCTTTCAATTGCTC |
| MADS7C2-F6 | ACTACTAGTGTCGACATTCATCCCTGAAGCACGTC |
| MADS7C2-R6 | TCTGAGCTCCTGCAGGTGGATGGAAGAACCCATTG |
| Actin-F | CCTGCTATGTACGTCGCCATC |
| Actin-R | CCGCAGCTTCCATTCCTATGA |
| UBQ10-F | TGGTCAGTAATCAGCCAGTTTGG |
| UBQ10-R | GCACCACAAATACTTGACGAACAG |
| HYG-F | GCTTTCAGCTTCGATGTAGGAGG |
| HYG-R | TTTCCACTATCGGCGAGTACTTC |
| Wx-2F | TTGCAGACAGGTACGAGAGG |
| Wx-2R | CTTCTCCAGGAATGACGGAT |
| Wx-10F | CCCCTCTCTCACCATTCCTT |
| Wx-10R | CGACATGGTGGTTGTCTAGC |
| SBEI-F | TGGCCATGGAAGAGTTGGC |
| SBEI-R | CAGAAGCAACTGCTCCACC |
| SBEIIb-F | ATGCTAGAGTTTGACCGC |
| SBEIIb-R | AGTGTGATGGATCCTGCC |
| SSSI-F | GGGCCTTCATGGATCAACC |
| SSSI-R | CCGCTTCAAGCATCCTCATC |
| AGP2b-F | AACAATCGAAGCGCGAGAAA |
| AGP2b-R | GCCTGTAGTTGGCACCCAGA |
| AGP2L-F | AGTTCGATTCAAGACGGATAGC |
| AGP2L-R | CGACTTCCACAGGCAGCTTATT |
| AGP3L-F | AAGCCAGCCATGACCATTTG |
| AGP3L-R | CACACGGTAGATTCACGAGACAA |
